# Development and validation of a multi-domain multimorbidity resilience index for an older population: results from the baseline Canadian Longitudinal Study on Aging

**DOI:** 10.1186/s12877-018-0851-y

**Published:** 2018-07-27

**Authors:** Andrew Wister, Scott Lear, Nadine Schuurman, Dawn MacKey, Barbara Mitchell, Theodore Cosco, Ian Fyffe

**Affiliations:** 1 Department of Gerontology, and Gerontology Research Centre, Simon Fraser University, 2800-515 Hastings Street, Vancouver, BC V6B 5K3 Canada; 2 Faculty of Health Sciences, Simon Fraser University, 8888 University Drive, Burnaby, BC V5A 1S6 Canada; 3 Department of Geography, Simon Fraser University, 8888 University Drive, Burnaby, BC V5A 1S6 Canada; 4 Department of Biomedical Physiology & Kinesiology, 8888 University Drive, Burnaby, BC V5A 1S6 Canada; 5 Departments of Gerontology and Sociology/Anthropology, Simon Fraser University, 2800-515 Hastings Street, Vancouver, BC V6B 5K3 Canada; 6 Gerontology Research Centre, Simon Fraser University, 2800-515 Hastings Street, Vancouver, BC V6B 5K3 Canada; 7 Gerontology Research Centre, Simon Fraser University, 2800-515 West Hastings St., Vancouver, V6B 5K3 Canada; 8 Department of Gerontology, Simon Fraser University, 2800-515 Hastings Street, Vancouver, BC V6B 5K3 Canada

**Keywords:** Multimorbidity resilience index, Multi-domain, Validation

## Abstract

**Background:**

Multimorbidity is recognized as a major public health issue that increases with age and affects approximately two-thirds of older people in Canada, the US, Australia and many European countries. This study develops and tests a three domain (functional, social and psychological) multimorbidity resilience composite index based on a previously developed lifecourse model of multimorbidity resilience, incorporating measures of adversity and positive adaptation. The criterion validity of the measure is demonstrated by means of an analysis of key outcome variables drawn from the literature.

**Methods:**

We used the baseline data from the Comprehensive Cohort of the Canadian Longitudinal Study on Aging. Associations of functional, social, psychological as well as total resilience with two health utilization and three illness context outcome variables were examined using logistic regression analyses, adjusted for age, gender, marital status, income, education, region, and number of chronic conditions.

**Results:**

The sample included all 6771 Canadian adults aged 65 or older (mean age 73.0, 57% women) who reported two or more of 27 possible chronic conditions. Total resilience was associated with: perceived health (OR = 1.68, CI 1.59–1.77); sleep quality (OR = 1.34, CI 1.30–1.38); perceived pain (OR = 0.80, CI 0.77–0.83); hospital overnight stays (OR = 0.87, CI 0.83–0.91); and emergency department visits (OR = 0.90, CI 0.87–0.94)., after adjusting for socio-demographic factors, and number of chronic conditions. These associations were similar for the unadjusted models, as well as for the functional, social and psychological resilience sub-indices.

**Conclusions:**

Combining components of adversity and positive adaptation within functional, social and psychological domains produces a measure of multimorbidity resilience that is associated with more positive health outcomes. Several implications of a composite multimorbidity resilience measure for clinical practice are identified. This measure can be replicated using measures found in other secondary health data sets. Future validation using longitudinal data is warranted.

**Electronic supplementary material:**

The online version of this article (10.1186/s12877-018-0851-y) contains supplementary material, which is available to authorized users.

## Background

Recently, there has been a growing interest in model developments aimed at understanding how individuals respond to illness-related adversities and regain a sense of wellness in their lives, termed *resilience* – the ability and resources needed to adapt and navigate stress-inducing experiences [1–3]. Although a broader literature has existed on resilience for decades applied to a variety of topics and populations [1, 4–7], research has been accumulating on specific substantive areas [8]. One of these is the application of resilience to older persons with more than one concurrent chronic disease – termed multimorbidity [8–12]. Multimorbidity has been shown to significantly increase with advanced age, and affects individuals adversely on many levels (i.e., physiological, psychological, social), making it appropriate for the application of resilience models. For instance, US clinical data show that 62% of persons aged 65–74, 75.7% aged 75–84, and 81.5% aged 85+ have 2 or more of 15 prevalent chronic illnesses [13]. Although variable depending on the type of data, population, and number of chronic illnesses, similar patterns have been observed in Canada and Australia [14]. Given that individuals with multiple chronic diseases are unlikely to completely overcome these adversities due to their permanence, and that a positive response may entail adaptation (coping) and/or only partly “bounce back” from their effects, we term this *multimorbidity resilience.*

Numerous measures of resilience have been developed and applied to different research questions and populations, but dominated by psychological measures that have been used to study mental health conditions and outcomes among children and among the general population [5, 7, 11]. Also, measures of resilience have been highly variable, depending on their theoretical and/or conceptual roots, methodological construction, and application, and typically are not adapted to an older population with unique multimorbidity illness contexts. This leaves significant research gaps, given that resilience measures are primarily psychological in nature (i.e., affective states), or qualitative, rather than covering measurable content domains based on underlying strengths and vulnerabilities associated with an aging and illness lens [5]. This paper operationalizes multimorbidity resilience as the combination of three domains: functional, social and psychological resilience, comprised of adversity and resilience components, and examines the criterion validity of these measures using key outcome measures of health care utilization and illness context among a vulnerable population of older individuals with multimorbidity.

There is likely no greater challenge to healthy aging and health care systems than the concurrent experience of multiple illnesses [15, 16]. Multimorbidity compounds the deleterious effects of living with individual chronic conditions by a synergistic exacerbating effect on symptom burden [17], lowering quality of life indicators, such as self-rated health and well-being [8, 18], and increasing complexity and cost of treatment [15, 16]. Yet, a gap in the literature is that we have a limited understanding of multimorbidity adaptation, self-care/coping, and healthy aging. The advancement of resilience concepts and the development of new measures and applications would assist in filling this void. This work is particularly relevant given the large and growing population of adults with multimorbidity who are aging during a period of escalating health care costs. For these reasons, there is a need to develop resilience measures that capture positive adaptation to illness contexts experienced among older populations.

Moreover, multimorbidity literature has predominantly focused on the pathogenic correlates, treatments, and disablement outcomes of multimorbidity, or of associated conditions or deficits, such as frailty [1, 6]. However, some individuals may possess important factors such as social support, economic resources and psychosocial strengths that may enable them to live well with and adapt to multiple chronic conditions [9, 10, 16, 19]. Although applications of resilience have often defined it in terms of recovery, the National Academy of Sciences has included adaptation as a central component that includes system reorganization, responses to stress, and social learning that can affect psychological resilience [20]. Applied to multimorbidity resilience, adaptation and coping may be more relevant than recovery, particularly given the chronic nature of multimorbidity in older age. Thus, there remains a significant gap in research that explicates the complexity of resilience types, processes, and determinants specific to the occurrence of chronic illness and disability in old age.

### Resilience, multimorbidity and aging

Resilience has been conceptualized in variable forms, including psychological, emotional, spiritual, physical/functional, economic, cultural, and social or ecological resilience [1–3, 6]. One popular measure with established psychometric properties is the Connor-Davidson Resilience Score (either the ten item or two item version) [4]. Similar to many resilience scales, such as the Brief Resilient Coping Scale [21], the Connor-Davidson Score measures the degree to which individuals perceive that they can overcome stress and adversity in life through a general set of questions [4]. However, this measure assumes that there is a singular concept of resilience [5] and it has not been specifically applied to multimorbidity among older people exposed to heightened health-related adversity. Therefore, this measure may be limited in this sub-field, since the items are not anchored in the experience of chronic illness or multimorbidity among older adults -- a condition that is slow in progression, long in duration, and typically limits function, productivity and quality of life [15, 16].

A common challenge is that any resilience measure entails combining levels of adversity with levels of positive response or adaptation, since resilience may be present but not activated without the occurrence of challenges. Some approaches have developed typologies based on ad hoc definitions of adversity thresholds and positive or negative responses [5]. Statistical or data-driven approaches have tended to use cross-sectional data, although some have used repeated-measure analyses of longitudinal data to identify a continuum of resilience, based on change in levels of adversity and adaptation [5]. Yet, there is no agreed upon approach to measuring resilience in the literature. While access to longitudinal data is preferable, there is a need for measurement development and testing that can be utilized in a broader set of studies and for specific populations [5, 7, 11].

Applications to multimorbidity among older adults requires consideration of several unique elements. The present research attempts to tap into resilience by first identifying a sub-population with exposure to adversity – multimorbid older adults; and second, by combining *both* adversity and adaptation (coping) factors into a multi-domain multimorbidity resilience index. A significant body of research has accumulated demonstrating the importance of health-related quality of life (HRQL) indicators of successful and healthy aging among persons with multimorbidity [7, 12]. A previous comprehensive systematic review of 112 articles (published between 1995 and 2015) supplemented with an additional 14 articles published since that time was conducted for the purpose of developing the Lifecourse Model of Multimorbidity Resilience (LMMR). In this study, we draw on the LMMR to identify a set of primary domains and associated measures of adversity and adaptation, and combine these into a composite index specifically applied to aging-related adversity and adaptation. The composite index (including sub-domains) is validated using outcomes identified in the multimorbidity literature.

The LMMR model connects several sources of resources embedded in the individual, family, community contexts, which can be harnessed in order to overcome the disablement processes of illness, and allow for individual reintegration and homeostasis. Adaptation/ resilience, as well as vulnerability/adversity processes, are understood as dynamic over an individual’s lifecourse. The LMMR provides an overarching framework and rationale for three resilience domains, each of which contains *both* adversity and adaptation (resilience) components [12]. 1) *Functional resilience* is deemed to be fundamental to aging well as it relates to the ability of a multimorbid individual to complete tasks of daily living, social roles, and remain physically active [1, 6]. Functional disability is a key aspect of the disablement process that can increase vulnerability and limit one’s ability to maintain daily activities of living, healthy living, as well as remaining engaged in community. 2) *Social resilience* can be understood as a multimorbid individual’s maintenance of positive social interaction, including community participation, as well as protecting against feelings of loneliness and experiencing social isolation that can result in negative adaptation. According to the LMMR, the successful activation of social resilience entails harnessing available resources, especially social support networks [9, 11]. An external activation of social resources may include support from a friend or family member, or the utilization of social capital derived from community participation. Social isolation, on the other hand, is expected to result in low levels of multimorbidity social resilience and integration [12]. 3) *Psychological resilience* pertains to the ability to mentally cope with stressors associated with multimorbidity. The degree to which individuals perceive stress in the face of multimorbidity, experience degrees of depression, and maintain psychological well-being represent aspects of this domain [11, 19]. This type of resilience draws from stress theory and the cognitive appraisal process [22], wherein stressfulness and challenges that are faced in old age due to episodic pain and disability can lead to the disruption of self-concept, health behaviours and health care decisions. Alternatively, feelings of well-being or satisfaction with life can result in internal activation of resources that can help individuals overcome adversity associated with chronic illness [19].

Multimorbidity resilience domains are assumed to be interrelated, fluid, and modifiable over the life course of individuals. Validation of a multimorbidity resilience index requires consideration of the unique characteristics of the individual domains as well as a composite index comprised of all content areas. Criterion validity is also required to examine correlations between the sub-indices, total index, and anticipated outcomes. Research indicates that a measure of multimorbidity resilience should be correlated with perceived health, as well as measures of health care utilization [9, 10, 16, 17].

## Methods

### Design and sample

This research utilizes a subset of the Comprehensive Cohort (see below) of the Baseline Wave of the Canadian Longitudinal Study on Aging (CLSA) dataset. Launched in 2010, this 20 year panel study of persons aged 45 to 85 has been funded primarily by the Canadian Institutes for Health Research (CIHR), Canada’s federal granting agency for health research. Data were being collected at baseline including biological, clinical, psychosocial and societal information that influence disease, health, and well-being [23]. The CLSA participants were randomly selected and invited to participate from the population aged 45 to 85 (excluding those living in institutions, full-time military, persons living on federal First Nations reserves and in the three northern territories), resulting in a total sample of 51,338. Inclusion criteria entailed being able to complete the interviews in English or French, live in the community, and be cognitively functional. The sampling method is the same as used by Statistics Canada for its Canadian Community Health Survey (CCHS) – Healthy Aging 2008/09 survey, and included participants from that study in its recruitment, supplemented with Provincial Health Registries, telephone sampling using random digit dialling, and the Quebec Longitudinal Study on Nutrition and Aging. The sample contains weights to adjust for sampling error and to produce a sample that is representative of the targeted Canadian population.

The CLSA is comprised of two cohorts of participants: 21241 Tracking Cohort participants and 30,097 Comprehensive Cohort participants, each of which can be used separately, since the Tracking Cohort collected only telephone-based data, whereas the Comprehensive Cohort included physiological data as well as a face-to-face interview [23].

The present research uses only the Comprehensive Cohort (*n* = 30,097), since several physiological measures are only available in this cohort [21]. Comprehensive participants were randomly selected within age/sex strata from within 25 km of dense population data sites, or within 50 km of data collection sites in areas with a lower population density. The 11 data collection sites for the CLSA are located in Victoria, BC; Vancouver, BC; Surrey, BC; Calgary, AB; Winnipeg, MB; Hamilton, ON; Ottawa, ON; Montreal, QC; Sherbrooke, QC; Halifax, NS; and St. John’s, NFLD.

Given our interest in developing and testing a multimorbidity resilience index among older adults, our study included only persons aged 65 or over who reported having two or more chronic conditions (*n* = 6771). Sample weights were used to correct for sampling error by age, gender, and geographic location. The sample self-reported two or more of 27 possible chronic conditions including Alzheimer’s disease, back problems, bowel incontinence, cancer, cataracts, diabetes, epilepsy, glaucoma, heart attack, heart disease, high blood pressure, irritable bowel syndrome, kidney disease, Parkinson’s disease, peripheral vascular disease, lung disease, macular degeneration, multiple sclerosis, osteoarthritis, osteoporosis, migraine headaches, rheumatoid arthritis, stroke, thyroid problem, transient ischemic attack, ulcer, and urinary incontinence. The validity and reliability of all relevant measures in the CLSA questionnaires, as well as references, can be found on the Data Portal of the CLSA web site (www.clsa-elcv.ca).

The independent variable with the largest amount of missing data was total household income (*n* = 610 or 9.0%). This variable was recoded using multiple imputation based on age, gender, education, and region. The remaining independent and dependent variables had minimal (under 3%) to no missing cases. Missing data for these variables were recoded to the respective means or modes. Analyses were replicated when removing missing data; therefore, only the analyses with all missing imputed data are presented.

### Measurement

#### Multimorbidity resilience index

A multimorbidity resilience index was created based on a composite (additive) index of three sub-indices representing functional, social, and psychological multimorbidity resilience domains. Each of these three sub-indices was, in turn, comprised of three index domain measures of adversity challenges and positive adaptation. Measures were coded into positive resilience, such as that higher scores reflect greater resilience.

#### Functional resilience variables

The three functional variables were the Older Americans Resources and Services

(OARS) Activities of Daily Living (ADL) Scale [24], the OARS Instrumental Activities of Daily Living (IADL) Scale [22], as well as the Summary Performance Score of functional ability scale [24]. The Summary Performance Score used in this study was calculated including a standing balance measure, a walk time measure, and a timed chair raise measure. Similar to this measurement construction, participants who completed these three tasks were assigned scores per task ranging from 1 to 4, which corresponded to statistical quartiles. Participants who did not complete a task were assigned a 0, with a range of 0 to 12 [25]. These lower extremity function tests directly measure physical challenge.

The OARS ADL Scale consisted of 7 items [24] of such tasks as eating and bathing. Each question was measured on a scale from 0 (completely unable) to 2 (completely able). Possible total scores range from 0 to 14, with higher scores indicating greater functional status. Similarly, the 7 item OARS IADL Scale also assesses functional ability [24]. Scores for the OARS IADL Scale questions also range from 0 to 2 and utilized the same coding scheme as above. These tasks are considered to be instrumental to daily living such as taking medicine and meal preparation, and reflect positive adaptation [6].

#### Social resilience variables

The three variables in this sub-index included the total Medical Outcomes Study (MOS) Social Support Survey [26], social participation, and a single item measuring perceived loneliness. The total MOS Social Support Survey instrument includes 19 items [26] consisting of the social support elements of emotional/informational support, affection support, tangible support, and positive social interaction. Each question ranges from 1 (none of the time) to 5 (all of the time). The scale has a range of 19–95 with higher scores indicating greater levels of social support. Social participation was a categorical measure developed by researchers at the CLSA. This variable asked the frequency of participation in activities with family or friends in the past 12 months. The answers ranged from “once a day”, “at least once a week”, “at least once a month”, “at least once a year”, to “never”. This question was recoded into “at least once a week or more” and “at least once a month or less”. The social support/participation measures are deemed to be significant resources for adaptation to multimorbidity A single item loneliness ordinal measure assessed how often a participant felt lonely over the past week. This categorical measure ranged from “all of the time, 5-7 days”, “occasionally, 3-4 days”, “some of the time, 1-2 days” to “rarely or never, less than 1 day”. Loneliness is associated with poor multimorbidity outcomes [12].

#### Psychological resilience variables

This sub-index included three variables: the Center for Epidemiological Studies Depression (CES-D) Scale [27], the Kessler Psychological Distress K10 Scale [28], and the Diener Satisfaction with Life Scale [29]. The CES-D Scale ranges from 0-28 and contains 10 questions on specific depression symptoms such as hopefulness, appetite and concentration. Each question has possible answers from 0 (rarely or none of the time, less than 1 day) to 3 (most or all of the time, 5–7 days). The Kessler Psychological Distress Scale [28] consists of 10 questions with a total range of 0–30. Answers to questions can range from 0 (never) to 3 (most of the time). The Depression and distress capture the psychological effects of illness adversity. The Diener Satisfaction with Life Scale [29] ranges from 5 to 35 with higher scores indicating greater life satisfaction. Individual questions range from 1 (strongly disagree) to 7 (strongly agree). It represents positive well-being and adaptation to illness [12, 22]. Although there is potential overlap of a few items in the depression and distress scales, these were deemed to have minimal effect on the index scores, given the number of items in the scales, and their unique constructs.

In order to standardize different measurement types and skewed distributions of measures, we employed an established and validated mapping system (converting all measures into scores between 0 and 10) using the normalization procedure for creating a frailty index [30], and applied to other areas such as an index of successful aging [31]. As shown in Table 1, ordinal measures were converted by dividing the number of responses into 10 proportionately. Continuous measures (after scale construction) were first converted into quartiles to address skewness, and then scaled to 0, 3.3, 6.7 and 10. The three sub-index scores were added and converted back to a range of 0 to 10 (by dividing by 3), and the total composite multimorbidity resilience index was an additive score of the three sub-index scores, and also converted to scores between 0 and 10 (by dividing by 3) for comparability. Higher scores indicated greater multimorbidity resilience.

**Table 1:** Total Resilience Scale Items, Values and Calculation

Item	Survey Question	Responses	Value	Score Calculation
Summary Performance Score		Lowest quartile	0	A
		Second lowest quartile	3.3	
		Second highest quartile	6.7	
		Highest quartile	10	
OARS ADL Scale		Lowest quartile	0	B
		Remainder	10	
OARS Instrumental ADL Scale		Lowest quartile	0	C
		Remainder	10	
Functional Resilience (FR)		Derived interval scale		(A + B + C) / 3 = FR
Satisfaction with Life Scale		Lowest quartile	0	D
		Second lowest quartile	3.3	
		Second highest quartile	6.7	
		Highest quartile	10	
Center for Epidemiologic Studies Depression Scale		Highest quartile	0	E
		Second highest quartile	3.3	
		Second Lowest quartile	6.7	
		Lowest quartile	10	
Kessler Psychological Distress Scale		Highest quartile	0	F
		Second highest quartile	3.3	
		Second lowest quartile	6.7	
		Lowest quartile	10	
Psychological Resilience (PR)		Derived interval scale		(D + E + F) / 3 = PR
MOS Social Support Total Scale		Lowest quartile	0	G
		Second lowest quartile	3.3	
		Second highest quartile	6.7	
		Highest quartile	10	
Loneliness	How often did you feel lonely in the past week?	All of the time (5–7 days)	0	H
		Occasionally (3–4 days)	3.3	
		Some of the time (1–2 days)	6.7	
		Rarely or never (< 1 day)	10	
Social Participation	Frequency of participation in family or friends activities out of the household	Never	0	I
		At least once a year	2.5	
		At least once a month	5.0	
		At least once a week	7.5	
		At least once a day	10	
Social Resilience (SR)		Derived interval scale		(G + H + I) / 3 = SR
Total Resilience (TR)		Derived interval scale		(FR + PR + SR)/3= TR

#### Criterion outcome variables

Review of the multimorbidity, resilience and aging literature in prior research revealed two primary areas in which we would anticipate associations with multimorbidity resilience. The first of these entails health care utilization. Indeed, extensive research has demonstrated that multimorbidity results in higher health care utilization, especially among older adults [15–17]. We therefore select two measures to assess criterion validity: emergency room visits; and hospital stays. These are expected to reveal inverse associations with the resilience indices.

The second set of variables (pain, perceived health and sleep quality) pertain to aspects of the illness context that affect quality of life, and are anticipated to be associated with multimorbidity resilience. Perception of pain is expected to have an inverse association with resilience, since it has been shown to be a debilitating consequence of multimorbidity [10, 16], with direct links to resilience concepts [2, 11]. Perceived health has been identified as one of the most consistent global measures of health and has been used in a multitude of studies of multimorbidity outcomes [14, 18], as well as multimorbidity measurement validation [1, 8]. Finally, sleep quality has also been associated with multimorbidity outcomes, and represents an important lifestyle factor predicted by deleterious illness experiences [32]. Perceived health and sleep quality are expected to have positive associations with the resilience indices. We use these five variables were used to assess criterion validity.

Health care utilization over the previous year was measured using: a) emergency department visitation, and b) overnight hospital admission coded no (0) and yes (1) in the CLSA dataset. Three ordinal outcome measures associated with illness context were dichotomized, given highly skewed distributions with few cases in some categories: c) pain was dichotomized into none/mild (0) and moderate/severe (1); d) perceived health was based on the single item question “how would you rate your health? This was scored into fair/poor (0) and good/very good/excellent (1); and e) sleep quality was based on the question: how satisfied are you with the quality of your sleep? This variable was coded: very dissatisfied/dissatisfied/neutral (0) and very satisfied/satisfied (1).

#### Covariates

Given the importance of several socio-demographic correlates of multimorbidity, we adjusted for the effects of age, gender, education level, total household income, marital status, and region in the logistic regression analyses [8, 13, 31]. In addition, the effects of number of chronic conditions (multimorbidity) was also adjusted in a separate model, given the potential association with resilience. The age variable ranged from 65 to 86. Gender was a dichotomous variable. The education level variable captured the highest degree, certificate, or diploma obtained by the participant. This variable was dummy coded with the level “no-post-secondary degree, certificate or diploma” as the control, as well as “education below Bachelors: trade certificate or diploma”, “Bachelor’s degree”, to “university degree or certificate above Bachelor’s degree” included as well. Total household income was also dummy coded with the level “less than $20,000” as the control; with “$20,000 to $49,999”, “$50,000 to $99,999”, “$100,000 to $149,999” and “$150,000 or above” as possible responses. The marital status variable was dichotomized from “single, never married or never lived with a partner”, “married/living with a partner in a common-law relationship”, “widowed”, “divorced”, and “separated” into “married/common-law” and “non-married/non-common-law” categories. Region was measured based on the five regions typically used in Canadian research: British “Columbia”, “Prairies”, “Ontario”, “Quebec”, and “Atlantic” regions. Number of chronic conditions were measured by counting illness categories (2 or more) based on 27 available categories.

### Data analyses

Intercorrelations among the resilience indices are presented first. We then employ logistic regression analyses for the dichotomized outcome measures, in order to assess associations with the four resilience indices (e.g. functional, social, psychological and total resilience), adjusting for the socio-demographic variables, as well as number of chronic conditions. The Statistical Package for the Social Sciences, Version 24, was used for the analyses.

## Results

### Intercorrelations of resilience indices

All frequencies and descriptive statistics are presented for the resilience measures, outcome variables, and covariates in Table 2. Table 3 shows the intercorrelations of the function, social, and psychological indices and the total resilience composite index, all of which are statistically significant (*p* < .001). The correlations among the sub-indices ranged between .20 (functional and social domains) and .46 (psychological and social domains), suggesting that they are capturing relatively distinct domains. The correlations between the sub-indices and the total composite index were .68, .69 and .82 for the social, functional and psychological domains, respectively. Given that the total index is a composite measure of the sub-indices, it is not surprising that these associations are stronger, with the largest association between the psychological resilience index and the total resilience index. The Cronbach’s alpha for the total resilience scale is .69.

**Table 2:** Descriptive Statistics (*n* = 6771)

Continuous Resilience Index Items	Range	Mean	Standard Deviation
Summary Performance Score	0 to 12	7.03	2.87
OARS ADL Scale	4 to 14	13.52	.91
OARS Instrumental ADL Scale	7 to 14	13.84	.60
Satisfaction with Life Scale	0 to 35	28.16	5.85
CES-D Depression Scale	0 to 28	5.41	4.51
Kessler Psychological Distress Scale	10 to 48	14.33	4.47
MOS Social Support Total Scale	0 to 64	51.15	11.02
Ordinal Resilience Index Items			Frequency (%)
Loneliness	All of the time (5–7 days)		164 (2.4)
	Occasionally (3–4 days)		651 (9.6)
	Some of the time (1–2 days)		1000 (14.8)
	Rarely or never (< 1 day)		4956 (73.2)
Social Participation	Never		55 (.8)
	At least once a year		510 (7.5)
	At least once a month		2558 (37.8)
	At least once a week		3362 (49.6)
	At least once a day		286 (4.2)
Resilience Index Variables	Range	Mean	Standard Deviation
Functional Resilience Index	0 to 10	7.09	2.61
Psychological Resilience Index	0 to 10	5.19	2.92
Social Resilience Index	0 to 10	6.65	1.88
Total Resilience Index	.28 to 10	6.31	1.83
Outcome Variables			Frequency (%)
Emergency Department Visitation	No		5252 (77.6)
	Yes		1519 (22.4)
Hospital Admission - Overnight	No		5909 (87.3)
	Yes		862 (12.7)
Pain	None/Mild		4901 (72.4)
	Moderate/Severe		1870 (27.6)
Perceived Health	Fair / Poor		755 (11.2)
	Good / Very Good / Excellent		6016 (88.8)
Sleep Quality	Very Dissatisfied / Dissatisfied / Neutral		2599 (38.4)
	Very Satisfied / Satisfied		4172 (61.6)
Continuous Independent Variables	Range	Mean	Standard Deviation
Age	65 to 86	73.16	5.68
Number of Chronic Conditions	2 to 16	4.03	1.93
Categorical/Ordinal Independent Variables			Frequency (%)
Gender	Female		3830 (56.6)
	Male		2941 (43.4)
Education	No post-secondary degree, certificate or diploma		2081 (30.7)
	Trade certificate or diploma		2101 (31.0)
	Bachelor’s degree		1270 (18.8)
	University degree or certificate above bachelor’s		1319 (19.5)
Household Income	Less than $20,000 per year		763 (11.2)
	$20,000 to $49,999		466 (6.9)
	$50,000 to $99,999		2219 (32.8)
	$100,000 to $149,999		2956 (43.7)
	$150,000 and over		367 (5.4)
Marital Status	Single / Widowed / Divorced / Separated		2304 (34.0)
	Married / Common-law		4467 (66.0)
Region	British Columbia		1341 (19.8)
	Prairies		1241 (18.3)
	Ontario		1532 (22.6)
	Quebec		1493 (22.1)
	Atlantic		1163 (17.2)

**Table 3:** Inter-Correlation Matrix for Multimorbidity Resilience Indexes, Weighted (*n* = 6771)

	Functional Resilience	Psychological Resilience	Social Resilience	Total Resilience
Functional Resilience	–	–	–	.69***
Psychological Resilience	.28***	–	–	.82***
Social Resilience	.20***	.46***	–	.68***

***p < .001

### Logistic regression analyses of resilience indices and outcome measures

Table 4 shows the logistic regression results of the three resilience sub-indices and the total resilience measure on the five outcome measures: a), unadjusted; b) controlling for the socio-demographic variables -- age, gender, marital status, education, income, and region; and 3) adjusting for the above socio-demographic variables as well as number of chronic conditions. The associations are shown both with and without number of chronic illnesses being adjusted to observe the effects of multimorbidity exceeding two chromic conditions, given the influence that multiple chronic conditions exerts on resilience [2, 14, 19] . In addition, relationships with the criterion outcome variables are statistically significant and in the hypothesized direction, based on the related literature (see Table 4). The associations are most pronounced for the total resilience measure for all five criterion outcome variables, although results for the sub-indices replicate findings for the total index (only results for the total index are discussed below). Confidence intervals around the estimated odds ratios (ORs) are very narrow, also indicating small standard errors.

**Table 4:** Logistic Regression for Multimorbidity Resilience Indices on Health Outcomes, Unadjusted, Adjusting for Socio-demographic Variables (Age, Gender, Marital Status, Education, Income, Region), and Number of Chronic Conditions (*n* = 6771)

	Functional Resilience				Psychological Resilience			
	Unadjusted	Socio-demographics	# Chronic Conditions	Final Model	Unadjusted	Socio-demographics	# Chronic Conditions	Final Model
	Exp(β) (C.I.)	Exp(β) (C.I.)	Exp(β) (C.I.)	β	Exp(β) (C.I.)	Exp(β) (C.I.)	Exp(β) (C.I.)	β
Emergency Department Visitation	.92 (.90 to .94)	.93 (.91 to .95)	.96 (.94 to .98)	−.04**	.92 (.90 to .94)	.92 (.90 to .94)	.94 (.92 to .96)	−.06***
Hospital Admission – Overnight	.88 (.85 to .90)	.88 (.85 to .90)	.91 (.88 to .94)	−.10***	.91 (.89 to .93)	.92 (.89 to .94)	.94 (.91 to .96)	−.07***
Pain	.84 (.83 to .86)	.85 (.84 to .87)	.89 (.87 to .91)	−11***	.84 (.82 to .86)	.86 (.84 to .87)	.88 (.86 to .90)	−.13***
Perceived Health	1.31 (1.28 to 1.35)	1.35 (1.31 to 1.39)	1.28 (1.24 to 1.31)	.24***	1.39 (1.34 to 1.43)	1.38 (1.34 to 1.43)	1.33 (1.29 to 1.38)	.29***
Sleep Quality	1.05 (1.03 to 1.07)	1.06 (1.04 to 1.08)	1.04 (1.02 to 1.06)	.04**	1.24 (1.22 to 1.26)	1.25 (1.22 to 1.27)	1.24 (1.22 to 1.27)	.22***
	Social Resilience				Total Resilience			
	Unadjusted	Socio-demographics	Chronic Conditions	Final Model	Unadjusted	Socio-demographics	Chronic Conditions	Final Model
	Exp(β) (C.I.)	Exp(β) (C.I.)	Exp(β) (C.I.)	β	Exp(β) (C.I.)	Exp(β) (C.I.)	Exp(β) (C.I.)	β
Emergency Department Visitation	.95 (.92 to .97)	.96 (.93 to .99)	.97 (.94 to 1.00)	−.03*	.86 (.84 to .89)	.87 (.84 to .90)	.90 (.87 to .94)	−.10***
Hospital Admission – Overnight	.92 (.89 to .96)	.95 (.91 go .99)	.97 (.93 to 1.01)	−.03*	.82 (.79 to .85)	.82 (.79 to .86)	.87 (.83 to .91)	−.15***
Pain	.91 (.88 to .93)	.93 (.91 to .96)	.95 (.92 to .98)	−.05**	.74 (.72 to .76)	.75 (.73 to .78)	.80 (.77 to .83)	−.23***
Perceived Health	1.22 (1.18 to 1.27)	1.21 (1.15 to 1.26)	1.18 (1.13 to 1.23)	.16***	1.72 (1.65 to 1.80)	1.82 (1.73 to 1.91)	1.68 (1.59 to 1.77)	.52***
Sleep Quality	1.14 (1.11 to 1.17)	1.16 (1.12 to 1.19)	1.15 (1.12 to 1.18)	.14***	1.29 (1.26 to 1.33)	1.35 (1.31 to 1.39)	1.34 (1.30 to 1.38)	.29***

*p < .05, ** *p* < .01, *** *p* < .001

We present three sets of odds ratios (ORs) and 95% confidence intervals for the total resilience index for the unadjusted and adjusted models. Unadjusted total resilience is associated with: perceived health (OR = 1.72, CI 1.65–1.80); sleep quality (OR = 1.29, CI 1.26–1.33); perceived pain (OR = 0.74, CI 0.72–0.76); hospital overnight stays (OR = 0.82, CI 0.79–0.85); and emergency department visits (OR = 0.86, CI 0.84–0.89).

After adjusting for the six socio-demographic variables, the associations between the total resilience index and the health outcomes were replicated, with only slight differences in ORs.These include: perceived health (OR = 1.82, CI 1.73–1.91); sleep quality (OR = 1.35, CI 1.31–1.39); perceived pain (OR = 0.75, CI 0.73–0.78); hospital overnight stays (OR = 0.82, CI 0.79–0.86); and emergency department visits (OR = 0.87, CI 0.84–0.90).

Finally, after also adjusting for number of chronic conditions, relationships were attenuated: perceived health (OR = 1.68, CI 1.59–1.77); sleep quality (OR = 1.34, CI 1.30–1.38); perceived pain (OR = 0.80, CI 0.77–0.83); hospital overnight stays (OR = 0.87, CI 0.83–0.91); and emergency department visits (OR = 0.90, CI 0.87–0.94). Results for the sub-indices were also consistent with the hypotheses, but were slightly weaker than for the total resilience measure (see Table 4).

Supplementary analyses were conducted focusing on subsets of three clusters of multimorbid conditions (vascular, osteoporosis, mental health) to decrease the variation of illnesses and symptomologies. The regression analyses replicated the above results (not presented).

## Discussion

This paper provided the rationale and development procedure for a composite index measuring multimorbidity resilience based on three sub-indices (measuring functional, social and psychological domains). Each of the domains were measured using three individual measures of both adversity and resilience, resulting in a continuous measure of total multimorbidity resilience. A standard method is used to standardize the distributions to create a composite index score, based on earlier work on the frailty index [30], and successful aging [31].

Results indicate that the total multimorbidity resilience index is inversely associated with emergency department visits, overnight hospital stats, and perceived pain. In addition, the total index is positively associated with perceived health and sleep quality. These associations are in the hypothesized directions and are replicated after adjusting for socio-demographic factors (age, gender, marital status, education, income and region), as well as number of chronic conditions. In order of strength, the total resilience index is associated with: perceived health; sleep quality; perceived pain; hospital overnight stays; and emergency department visits. These criterion variables represent important outcomes of multimorbidity experience among older adults with important health and health care implications.

Comparisons of the criterion validity analyses using our composite resilience index and other resilience measures provides additional evidence of criterion-related validity. Although the available literature focusing on chronic illness, especially among older adults is sparse, there are some useful comparative studies. The Brief Resilient Coping Scale (BRCS), a 4-item measure of tendencies to cope with stress in an adaptive manner, has been shown to have adequate internal consistency (baseline Cronbach’s alpha = .64) and test-retest reliability (.71) [21]. Using two samples of persons (mean ages 46 and 58, respectively) with rheumatoid arthritis, the BRCS did not correlate with age, employment stats, length of time since diagnosis, pain, or fatigue in either sample at baseline [21]. However, predictable correlations were found with pain coping behaviours and psychological well-being, the former of which is consistent with the multimorbidity resilience index.

In a study of general resilience (measured by cumulative lifetime adversity, social support and mastery) over a two-year period based on persons aged 50–70 drawn from the US Health and Retirement Survey, researchers supported an inverse association with hospital utilization OR = 0.75, CI 0.64–0.86), and a positive association with self-rated health (OR = 1.49, CI 1.17–1.88), after adjusting for socio-demographic and lifestyle covariates [32]. These associations are virtually identical (but slightly weaker), to the ones found in our CLSA study using the composite multimorbidity resilience index for overnight hospital admissions (OR = .87, CI .83–.91), and perceived health (OR = 1.68, CI 1.59–1.77), after adjusting for all covariates.

Another study of general resilience (measured as a stressful event within 5 years, level of stressfulness and level of recovery), analyzed a sample of 546 non-disabled older adults, i.e. who did not require personal assistance, aged 70+ living in the US [33]. While non-disabled, 56% of their sample had two or more chronic conditions, making them comparable to the CLSA sample. The researchers found associations between their six-item resilience measure and functional status, depression and self-rated health (SRH). Of particular relevance, their findings for SRH were consistent with ours (OR = 1.38, CI 1.01–1.79) after adjusting for socio-demographic and functional measures. In addition, other studies have shown support for associations between resilience and pain, as well as sleep, although not directly comparable to the CLSA sample [2, 34]. Taken together, review of available studies show that our results of the criterion validity outcome analyses using the total multimorbidity resilience index are comparable.

There are a number of limitations of this research. First, cross-sectional study designs can not capture change in resilience levels and outcomes, which is needed to substantiate this work. Further, we are unable to draw conclusions regarding the direction of causality due to the temporal nature of these data. As more waves of the CLSA become available, the resilience indices can be validated with longitudinal data. Second, there are other methods of standardizing the resilience sub-indices and total index scores with respect to weighting and item inclusion. The current method is informed by the procedure used in the development of the Frailty Index [30] and Successful Aging Index [31]. Third, some level of granularity is lost when converting continuous scores into quartiles. Fourth, some of the items in the composite resilience index overlap (especially, the Kessler Psychological Distress K10 scale, the CES-D scale, and the loneliness item). The large number of items and scoring system mitigates the extent to which this biases the results.

## Conclusions

This study developed a new multimorbidity resilience index comprised of functional, social and psychological domains with measures of adversity and adaptation. The criterion validation of the index and comparisons with similar studies provides initial support for this new measure. Although the study does not account for the stage of the comorbidity and its severity, the consistency of associations with hypothesized outcomes has several implications. First, a composite resilience index can be used to identify individuals at higher risk of emergency department visits and/or hospitalization, as well as perceptions of health, pain and sleep quality. Second, a composite resilience measure can be extended to other areas of risk, such as injury and falls. Third, identification of individuals at lower levels of resilience can be helpful in interventions aimed at improving independent community living. All of these clinical implications can potentially lower health care costs and extend longevity.

Further confirmatory research is needed to validate the resilience indices using other known data sets, such as the US Health and Retirement Study. In addition, these measures need to be incorporated into explanatory and predictive models in order to identify and compare determinates and outcomes, especially using longitudinal data sources. Research is also warranted to establish the full usefulness of this measure among different populations (e.g., race, ethnicity, socio-economic status, etc.), as well as applications to relevant clinical settings.

## Additional file

Additional file 1: Universities and organizations participating in the harmonized CLSA ethics. (DOCX 13 kb)

## Abbreviations

ADL: Activities of Daily Living
CCHS: Canadian Community Health Survey
CED-D: Center for Epidemiological Studies Depression
CIHR: Canadian Institutes of Health Research
CLSA: Canadian Longitudinal Study of Aging
HRQL: Health related quality of life
IADL: Instrumental activities of daily living LMMR – Lifecourse model of multimorbidity resilience
MOS: Medical Outcomes Study
OARS: Older Americans Resources and Services
SFU ORS: Simon Fraser University, Office of Research Services
