# Supplementary material for: Development and validation of a multi-domain multimorbidity resilience index for an older population: results from the baseline Canadian Longitudinal Study on Aging
Source: BMC Geriatr. 2018 Jul 27;18:170. doi: 10.1186/s12877-018-0851-y (PMC6062931; doi:10.1186/s12877-018-0851-y)
Supplement: Supplementary file 1 — Universities and organizations participating in the harmonized CLSA ethics. (DOCX 13 kb) [file 12877_2018_851_MOESM1_ESM.docx]

|  | University/Health Authority/Institute | Committee Name |
| --- | --- | --- |
| 1 | Hamilton Health Sciences/McMaster REB | (Hamilton Integrated Research Ethics Board – HiREB) |
| 2 | Universite de Sherbrooke | Bureau des plaints et de la qualité des services  CIUSS de l’Estrie – CHUS |
| 3 | Dalhousie University | Office of Research Ethics Administration |
| 4 | University of Manitoba | Bannatyne Campus Research Ethics Board |
| 5 | McGill University | McGill Institutional Review Board |
| 6 | Memorial University of Newfoundland | Health Research Ethics Authority |
| 7 | University of Victoria | Human Research Ethics Board |
| 8 | Elisabeth Bruyere Research Institute of Ottawa | Bruyere Research Ethics Board |
| 9 | University of British Columbia | Office of Research Ethics |
| 10 | Island Health – formerly Vancouver Island Health Authority (VIHA) | Research Ethics Office |
| 11 | Simon Fraser University | Office of Research Ethics |
| 12 | Calgary CHREB | Conjoint Health Research Ethics Board |
| 13 | PEI Health | PEI Research Ethics Board – Health Association of PEI |
